# Risk Factors of Fecal Toxigenic or Non-Toxigenic *Clostridium difficile* Colonization: Impact of Toll-Like Receptor Polymorphisms and Prior Antibiotic Exposure

**DOI:** 10.1371/journal.pone.0069577

**Published:** 2013-07-25

**Authors:** Yuan-Pin Hung, Hsiao-Ju Lin, Tai-Chieh Wu, Hsiu-Chuan Liu, Jen-Chieh Lee, Chih-I Lee, Yi-Hui Wu, Lei Wan, Pei-Jane Tsai, Wen-Chien Ko

**Affiliations:** 1 Department of Internal Medicine, Tainan Hospital, Department of Health, Executive Yuan, Tainan, Taiwan; 2 Department of Experiment and Diagnosis, Tainan Hospital, Department of Health, Executive Yuan, Tainan, Taiwan; 3 Department of Internal Medicine, National Cheng Kung University Hospital, Tainan, Taiwan; 4 Graduate Institute of Clinical Medicine, National Health Research Institutes, Tainan, Taiwan; 5 Department of Medical Laboratory Science and Biotechnology, National Cheng Kung University, Medical College, Tainan, Taiwan; 6 Department of Internal Medicine, PingTung Christian Hospita, PingTung, Taiwan; 7 School of Chinese Medicine, China Medical University, Taichung, Taiwan; 8 Center for Infection Control, National Cheng Kung University Hospital, Tainan, Taiwan; 9 Department of Medicine, National Cheng Kung University Medical College, Tainan, Taiwan; Charité, Campus Benjamin Franklin, Germany

## Abstract

**Background:**

This study is to investigate the significance and risk factors of fecal toxigenic (tCdC) or non-toxigenic *Clostridium difficile* colonization (ntCdC) among hospitalized patients.

**Methods:**

Adults admitted to medical wards in a district hospital between January 2011 and June 2012 were enrolled, and those with a history of colectomy, *C. difficile* fecal colonization or infection or receipt of either metronidazole or oral vancomycin within 3 months, were excluded. Stools collected within 48 hours after admission and every week during hospitalization were cultured for *C. difficile*.

**Findings:**

Among the 441 enrolled patients, 84 (20.0%) had CdC at initial screening, including 58 (13.2%) with tCdC and 26 (6.8%) with ntCdC. Among patients with initial negative fecal screening for CdC, it took an average of 70.6 days or 66.5 days to develop tCdC or ntCdC during the study period. Finally 78 (17.7%) had tCdC and 34 (7.7%) had ntCdC. During the follow-up period, the patients with tCdC had a higher risk of CDAD (11/79, 14.1%) than those without CdC (3/328, 0.9%) and those with ntCdC (0/34, 0%) (*P*<0.001). In multivariate analysis, the TLR4 rs1927914 polymorphism (GG genotype) (odds ratio [OR] 4.4, 95% confidence interval [CI] 1.6–11.8, *P* = 0.003) and recent cefepime therapy (OR 5.3, 95% CI 2.1–13.2, *P*<0.001) were independently associated with tCdC, whereas recent cefuroxime (OR 11.7, 95% CI 2.3–60.2, *P* = 0.003) and glycopeptide therapy (OR 10.9, CI: 2.1–57.2, *P* = 0.005) associated with ntCdC.

**Conclusion:**

The incidence of CDAD is highest in patients with tCdC and lowest in patients with ntCdC, and the TLR4 rs1927914 polymorphism GG genotype and recent cefepime therapy were independently associated with tCdC.

## Introduction

*Clostridium difficile* is a major cause of nosocomial antibiotic-associated diarrhea due to the production of toxins A and B. *C. difficile* isolates that are capable of producing toxins A and B are regarded as toxigenic. The clinical features typically include diarrhea, lower abdominal pain and systemic symptoms, such as fever, anorexia, nausea and malaise, but they can range from mild diarrhea to pseudomembranous colitis or toxic megacolon. *C. difficile* is frequently transmitted in healthcare settings via the hands of healthcare workers, and thus, *C. difficile-*associated diarrhea (CDAD) is an important infection control issue. The incidence of *C. difficile* infection was 42.6 cases per 100,000 patient-days and was increasing in recent years in Taiwan in our previous retrospective study [1]. Advanced age, the use of antibiotics, prolonged hospitalization, the presence of comorbidity with functional impairment or immune gene polymorphism (such as IL-8) are associated with increased rates of *C. difficile* infection and disease recurrence [2], [3], [4]. The interaction of *C. difficile* with the innate immune system likely plays a major role in the pathogenesis of colitis or pseudomembranous disease, yet it remains an area that has received little attention.

Toxigenic *C. difficile* colonization (tCdC) has been reported as an independent factor of subsequent development of CDAD [5]. However, the variables associated with tCdC were not reported before. Nontoxigenic *C. difficile* colonization (ntCdC) was noted to prevent CDAD in retrospective studies, and the administration of nontoxigenic *C. difficile* isolates has been used to prevent CDAD in animal studies [6], [7]. However, nontoxigenic *C. difficile* isolates have been discovered in the unformed stool of hospitalized patients developing diarrhea in hematology/oncology wards [8]. The clinical relevance of ntCdC to diarrhea is not clear, and risk factors for the acquisition of tCDC or ntCdC are ambiguous due to the lack of prospective studies.

Toll-like receptors (TLRs) are a class of single membrane-spanning receptors and are the major component of the antimicrobial armamentarium of innate immune cells that recognize invading organisms [9], [10]. Among the TLR family members, TLR2 recognizes multiple components of several bacterial cell walls, including peptidoglycans and lipoproteins in the cell walls of several bacteria and mycoplasmas [11], [12]. Patients with TLR2 single nucleotide polymorphisms (SNPs) have been associated with many gram-positive infections, such as *Bacillus subtilis, Staphylococcus aureus, Streptococcus pneumoniae*, and *Listeria monocytogenes* [11]. TLR4 is a major receptor for lipopolysaccharide (LPS), a component of gram-negative bacterial cell walls [12], and it is associated with gut innate immunity. Patients with TLR4 polymorphisms were more likely to have intestinal infections due to gram-negative organisms, Crohn’s disease, or ulcerative colitis [11], [12]. Both TLR2 and TLR4 signaling result in the activation of inflammatory pathways involving nuclear factor-kappa B (NF-κB) [13], and thus NF-κB polymorphisms play a critical role in the activation of innate immunity [13]. However, the role of TLR2, TLR4 or NF-κB polymorphism in the development of CdC or CDAD is not defined. Therefore, the aims of the present study are to delineate the clinical significance of tCdC and ntCdC and to investigate the association of certain host factors, including age, comorbidities, TLR2, TLR4 or NF-κB polymorphism, and prior antibiotic use, with CdC or ntCdC.

## Materials and Methods

A prospective investigation was conducted in the medical wards of the Tainan Hospital, Department of Health, Executive Yuan, a district hospital in southern Taiwan, from January 2011 to June 2012, and had included the study population in our previous report [5]. The study was approved by the institutional review board of Tainan Hospital, Department of Health, Executive Yuan, and written informed consent was obtained from all patients. The inclusion criteria included both males and females, aged at least 18 years who were admitted to medical wards with expected hospital stays of at least 5 days. A patient with fecal CdC or CDAD within previous three months, metronidazole or oral vancomycin therapy within previous three months, colectomy, CDAD on admission, or fecal colonization or infection due to non-*difficile Clostridium* spp. that had been reported to be related to diarrhea, was excluded [14], [15], [16]. When the patients were re-admitted, the observation clock was restarted and stool was collected again. The enrolled patients were followed up at each hospitalization between January 2011 and June 2012. The end of follow-up was the last hospitalization before June 2012. We recorded general condition and medication history between hospitalizations. The follow-up duration was estimated from the day of inclusion to the discharge from the last hospitalization before June 2012. Some of the patients had several hospitalizations during the follow-up period.

Diarrhea was defined as a change in bowel habit with more than three unformed bowel movements per day for at least 2 days. Information about the patient’s status prior to admission, including comorbid conditions or a history of CdC or CDAD, was prospectively queried. Clinical data, including age, nasogastric tube use or underlying disease, were recorded based on the first admission of each patient. The Charlson comorbidity index was used to estimate the severity of underlying diseases [17]. Chronic kidney disease (CKD) was defined as an estimated glomerular filtration rates (GFR) <60 mL/min/1.73 m^2^ for at least three months [18]. All antibiotics prescribed within one month before CDAD or at the end of follow-up were recorded. The cephalosporin category included cefazolin, cefuroxime, 3^rd^ generation cephalosporins (ceftriaxone or ceftazidime), and cefepime. The category of penicillins other than piperacillin/tazobactam included the penicillin derivatives (penicillin, oxacillin or piperacillin) and beta-lactam/beta-lactamase inhibitors (amoxicillin/clavulanic acid or ampicillin/sulbactam). Carbapenems included imipenem/cilastatin, meropenem and ertapenem. The glycopeptide category was composed of vancomycin and teicoplanin.

Three TLR2 SNPs (rs1898830, rs3804099, and rs7656411) [19], two TLR4 SNPs (rs10983755 and rs1927914) [20], [21], and the NF-κB SNP (−94insertion/deletion ATTG) [22], which are highly prevalent in the Chinese population, were selected for investigation. The patients’ DNA was extracted using a kit (Geneaid Genomic DNA Mini Kit) according to the manufacturer’s instructions. The TLR2, TLR4 and NF-κB polymorphisms were studied using real-time quantitative PCR (Applied Biosystems) using the TaqMan® Pre-Designed SNP Genotyping Assays. The probe sequences used are described in Table S1.

Fecal samples collected within 48 hours after admission and every week during hospitalization were sent for stool cultures. Stool samples plated on cycloserine-cefoxitin-fructose agar (CCFA) were cultured under anaerobic conditions. If the patient had been readmitted, the stool samples were collected again, and they were repeatedly collected every week during the hospitalization. When a hospitalized patient developed diarrhea, the stool culture was repeated. Stool samples were transported to the clinical laboratory within less than one hour of collection.

As previously described, asymptomatic *C. difficile* colonization was defined as a positive stool culture for *C. difficile* in the absence of diarrhea [2]. If the fecal *C. difficile* isolate was toxigenic or non-toxigenic, as evidenced by the presence or absence of *tcdB* by PCR, such an event was defined as tCdC or ntCdC, respectively. A patient with *tcdB*-carrying *C. difficile* isolated in the feces, in the presence of diarrhea without an alternative explanation, was regarded to have CDAD, which was modified as previously described [2]. All enrolled patients were followed until discharge or death. The primary outcome was the occurrence of CDAD, and the secondary outcome was the crude mortality rate.

Statistical analysis was performed using the statistical software (SPSS, version 13.0). Continuous data were expressed as the means ± standard deviations. The χ^2^ test or Fisher’s test was used for categorical variables, and the Student *t*-test for continuous variables. A two-tailed *P* value of less than 0.05 was considered to be statistically significant. The parameters with *P* values less than 0.05 in the univariate analysis were entered into a multivariate analysis with a binary logistic regression model. The Bonferroni correction for multiple testing was applied.

## Results

### Study Population

**Figure 1:**
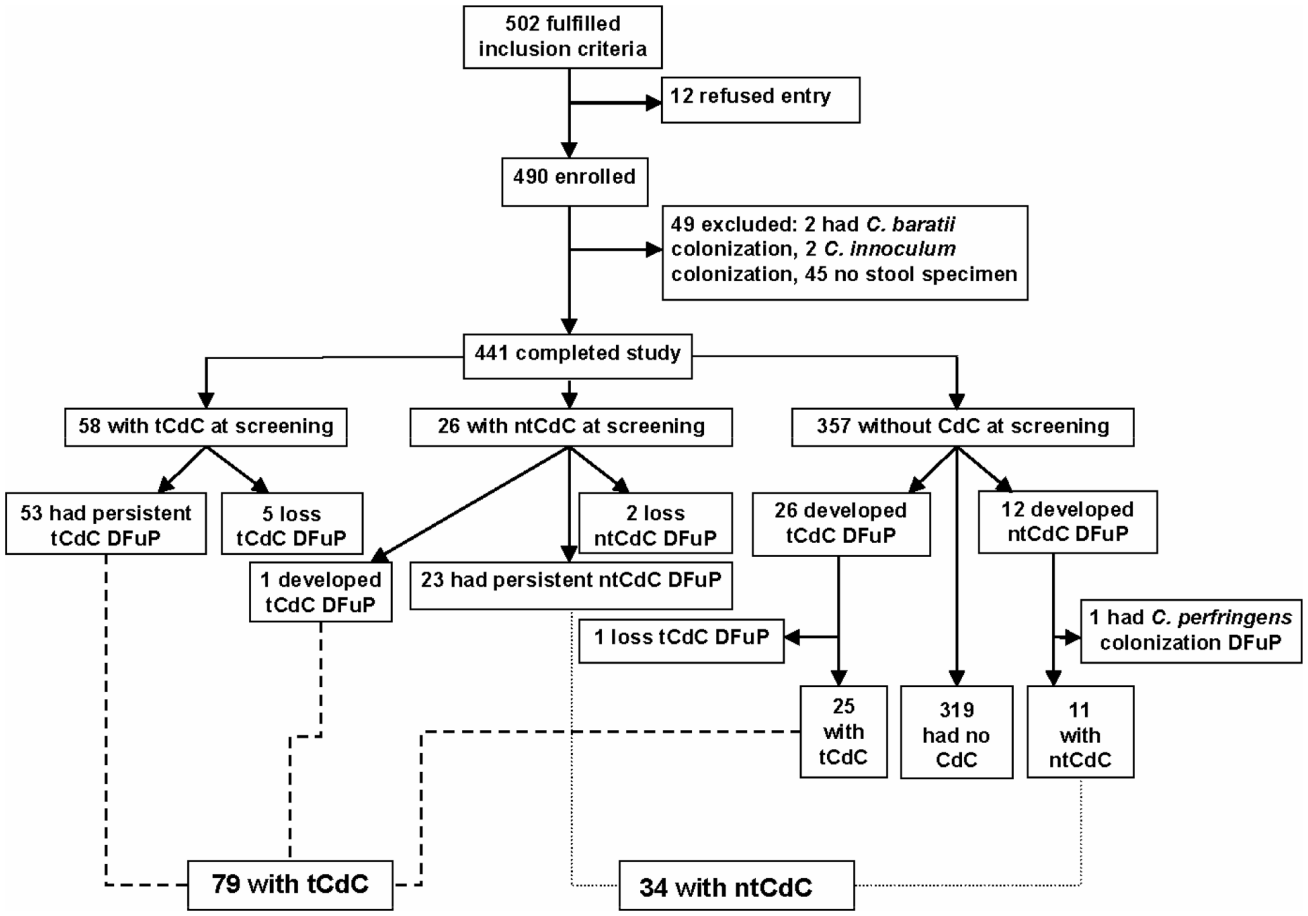
Results of screening for fecal toxigenic and nontoxigenic *Clostridium difficile* colonization at the study hospital. Note: tCdC = toxigenic *Clostridium difficile* colonization; ntCdC = nontoxigenic *C. difficile* colonization; DFuP = during follow-up period.

A total of 502 patients were evaluated, and 12 refused to participate in the study. Thus, 490 patients were enrolled in the prospective survey, but 49 were excluded due to fecal colonization by *C. baratii* (2 patients) or *C. innoculum* (2), or no stool specimen available within 48 hours after admission (45) (Fig. 1). Finally, an overall total of 441 patients with a slight male predominance (236, 53.5%) completed the study. Fifty five (12.5%) patients had more than two hospitalizations during the study period, and the majority (53, 96.4%) of those with multiple hospitalizations resided in nursing home, where medical information was recorded in details and available at admission. Of the 441 patients, 84 (20.0%) had CdC at screening, including 58 (13.2%) with tCdC and 26 (6.8%) with ntCdC. During the study period, 5 (8.6%) of the 58 patients with tCdC at screening became free of tCdC, and 2 (7.7%) of the 26 with ntCdC at screening became free of colonization. One of the 26 patients with ntCdC developed tCdC. One patient developed *C. perfringens* colonization 60 days after ntCdC, but lost the colonization 7 days later (Fig. 1).

Of the 357 patients free of CdC at the initial stool screening, 26 (7.3%) patients developed tCdC and 12 (3.4%) developed ntCdC during the study period. Therefore, the estimated incidence density or cumulative incidence of tCdC was 8.48 cases/10,000 patient-days or 73.0/1,000 patients, and for ntCdC the incidence was 3.91 cases/10,000 patient-days or 34 cases/1,000 patients. It took an average of 70.6±79.5 (6–331, medium 37) days or 66.5±81.8 (6–268, median 26) days after the initial negative fecal screening to develop tCdC or ntCdC. The patients with tCdC had longer follow-up durations than those without CdC (156.4 *vs.* 79.2 days, *P* = 0.05).

### Impact of CdC

Among the 441 enrolled patients, 78 (17.7%) had tCdC and 34 (7.7%) had ntCdC at the end of the follow-up period. During the follow-up, 328 (74.4%) patients were free of CdC. The patients with tCdC (8/79, 10.1%) or ntCdC (5/34, 14.7%) more often experienced diarrhea than those without CdC (13/328, 4.0%). However, 5 (14.7%) of the 34 patients with ntCdC experienced diarrheal illness not related to *C. difficile*, with evidence of no fecal growth of *C. difficile* (2 cases) or persistent fecal growth of non-toxigenic *C. difficile* (3 cases). During the follow-up period, the patients with tCdC had a higher risk of CDAD (11/79, 14.1%) than those without CdC (3/328, 0.9%) and those with ntCdC (0/34, 0%) (*P*<0.001). In contrast, there was no patient with ntCdC who developed CDAD. However, crude mortality rates were similar among patients with tCdC (14.1%, 11/79), ntCdC (17.6%, 6/34) and no CdC (12.6%, 41/328). Overall, 14 (3.2%) of the 441 patients experienced CDAD, resulting in a CDAD incidence density or cumulative incidence of 4.57 cases/10,000 patient-days or 31.2 cases/1,000 patients. Of the 14 patients who developed CDAD, 8 (57.1%) were males, and the mean age was 74.0±12.3 years. The mean time to diarrhea was 29.2±35.4 days, and the mean hospitalization duration was 55.1±37.2 days. Eleven of the 13 patients had preexisting tCdC, including 3 patients who were free of CdC at the initial stool screening but developed tCdC during the follow-up and 8 who had CdC at the initial stool screening. Three (21.4%) of the 14 patients expired but not directly associated with CDAD.

Of the 357 patients without CdC at the screening, 8 developed CDAD (including 3 who developed tCdC during the follow-up and 5 without preexisting CdC), with an incidence density or cumulative incidence of CDAD of 1.93 cases/10,000 patient-days or 15.2 cases/1,000 patients and 58 with tCdC for an incidence of 20.46 cases/10,000 patient-days or 101.3 cases/1,000 patients. There were no patients experiencing recurrent CDAD during the study period.

### Risk Factors of tCdC or ntCdC

To assess the risk factors and genetic polymorphisms related to developing tCdC or ntCdC in the patients, the 357 patients without CdC at the initial screening were analyzed. There were no differences in terms of underlying diseases or the Charlson comorbidity indexes among the patients with tCdC, ntCdC and those without CdC (Table 1). Blood samples of 347 (97.2%) patients were available for the study of genetic polymorphism. The TLR4 rs1927914 polymorphism GG genotype was more often noted in those with tCdC than those without CdC (34.8%, 8/23 *vs.* 13.7%, 43/313; *P* = 0.02). There was no correlation between patients with the TLR2 polymorphisms (rs1898830, rs3804099, and rs7656411), the TLR4 rs10983755 polymorphism, and the NF-κB −94insertion/deletion ATTG polymorphism and CdC (Table 2).

**Table 1:** Comparisons of 357 patients without fecal *Clostridium difficile* colonization (CdC) at admission, stratified by the presence or absence of toxigenic or nontoxigenic CdC in the feces during hospitalization.

Character	Total,	No CdC,	Toxigenic	Nontoxigenic	*P* value		
	n = 357	n = 321	CdC, n = 25	CdC, n = 11	a	b	c
Male gender	195 (54.6)	176 (54.8)	15 (60.0)	4 (36.4)	0.68	0.36	0.28
Age (mean ± standard deviation [SD])	72.7±15.8	72.8±15.7	71.3±15.9	74.6±20.9	0.65	0.70	0.60
Nasogastric tube feeding	179 (50.1)	160 (49.8)	13 (52.0)	6 (54.5)	1.00	1.00	1.00
Charlson comorbidity index (mean ± SD)	1.7±1.5	1.8±1.5	1.3±1.3	1.0±1.5	0.16	0.10	0.52
Underlying disease							
Hypertension	171 (47.9)	158 (49.2)	7 (28.0)	6 (54.5)	0.06	0.77	0.15
Stroke history	144 (40.3)	133 (41.4)	6 (24.0)	5 (45.5)	0.10	0.77	0.25
Diabetes mellitus	126 (35.3)	116 (36.1)	9 (36.0)	1 (9.1)	1.00	0.11	0.13
Chronic kidney disease	45 (12.6)	42 (13.1)	1 (4.0)	2 (18.2)	0.34	0.65	0.22
Chronic obstructive pulmonary disease	43 (12.0)	40 (12.5)	3 (12.0)	0	1.00	0.37	0.54
Congestive heart failure	25 (7.0)	20 (6.2)	3 (12.0)	2 (18.2)	0.23	0.16	0.63
Malignancy	22 (6.2)	21 (6.5)	1 (4.0)	0	1.00	1.00	1.00
Liver cirrhosis	4 (1.1)	4 (1.2)	0	0	1.00	1.00	-
Follow-up duration, days (mean ± SD)	85.9±126.9	79.2±121.7	156.4±173.6	121.0±110.4	0.05	0.25	0.47
Community stay within the last month ofcolonization or follow-up	277 (77.6)	251 (78.2)	18 (72.0)	8 (72.7)	0.46	0.71	1.00

Data are no. (%) of patients, unless otherwise indicated.

a toxigenic CdC *vs.* no CdC;

b nontoxigenic CdC *vs.* no CdC;

c toxigenic CdC *vs.* nontoxigenic CdC.

**Table 2:** Correlation of fecal *Clostridium difficile* colonization (CdC) at admission among 347 patients with NF-kB, TLR-2 or TLR-4 polymorphism.

Characters	Total,n = 347	No CdC, n = 313	Toxigenic CdC, n = 23	NontoxigenicCdC, n = 11	*P* values		
					a	b	c
**NF-kB polymorphism**					1.00	0.36	0.49
ins/ins genotype	120 (33.6)	109 (34.8)	8 (34.8)	3 (27.3)			
ins/del genotype	152 (42.6)	135 (43.1)	10 (43.5)	7 (63.6)			
del/del genotype	75 (21.0)	69 (22.0)	5 (21.7)	1 (9.1)			
**TLR-2 polymorphism**							
rs1898830					0.27	0.57	0.68
AA genotype	108 (30.3)	100 (31.9)	6 (26.1)	2 (18.2)			
GA genotype	175 (49.0)	153 (48.9)	15 (65.2)	7 (63.6)			
GG genotype	64 (17.9)	60 (19.2)	2 (8.7)	2 (18.2)			
rs3804099					0.08	0.60	0.22
TT genotype	166 (46.5)	145 (46.3)	15 (65.2)	6 (54.5)			
TC genotype	153 (42.9)	143 (45.7)	5 (21.7)	5 (45.5)			
CC genotype	28 (7.8)	25 (8.0)	3 (13.0)	0 (0)			
rs7656411					0.28	0.94	0.55
TT genotype	94 (26.3)	82 (26.2)	9 (39.1)	3 (27.3)			
TG genotype	174 (48.7)	160 (51.1)	8 (34.8)	6 (54.5)			
GG genotype	79 (22.1)	71 (22.7)	6 (26.1)	2 (18.2)			
**TLR-4 polymorphism**							
rs10983755					0.15	0.30	0.06
GG genotype	204 (57.1)	186 (59.4)	9 (39.1)	9 (81.8)			
GA genotype	118 (33.1)	105 (33.5)	11 (47.8)	2 (18.2)			
AA genotype	25 (7.0)	22 (7.0)	3 (13.0)	0 (0)			
rs1927914					0.02	0.49	0.28
AA genotype	136 (38.1)	128 (40.9)	5 (21.7)	3 (27.3)			
AG genotype	159 (44.5)	142 (45.4)	10 (43.5)	7 (63.6)			
GG genotype	52 (14.6)	43 (13.7)	8 (34.8)	1 (9.1)			

Data are no. (%) of patients, unless otherwise indicated.

a toxigenic CdC *vs.* no CdC;

b nontoxigenic CdC *vs.* no CdC;

c toxigenic CdC *vs.* nontoxigenic CdC.

The association of recent antibiotic exposure with a patient’s developing tCdC or ntCdC was evaluated among the individuals without CdC at the initial screening. Patients who had received more than one class of antibiotics (OR 3.75, 95% CI 1.46–9.63, *P* = 0.006), particularly a cephalosporin plus a penicillin (OR 3.92, 95% CI 1.51–10.15, *P* = 0.09) or a cephalosporin plus a carbapenem (OR 3.72, 95% CI 1.60–8.62, *P* = 0.03), had a higher risk of tCdC compared to no CdC. On the other hand patients who had received more than one class of antibiotics (OR 5.33, 95% CI 1.13–25.04, *P* = 0.03), particularly a penicillin plus a glycopeptide (OR 7.15, 95% CI 1.73–29.54, *P* = 0.02) or a cephalosporin plus a carbapenem (OR 3.94, 95% CI 1.16–13.38, *P* = 0.03), had a higher risk of ntCdC compared to no CdC. Then we analyze the impact of individual antibiotic exposure on CdC (Table 3). Recent prescriptions for cefuroxime (36.4 *vs*. 9.3%, *P* = 0.02) and intravenous glycopeptide (45.5 *vs*. 15.0%, *P* = 0.02) were more frequent in the subjects with ntCdC than in those without CdC, and in contrast, recent cefepime therapy was associated with tCDC (48.0 *vs*. 17.4%, *P* = 0.001). There was no correlation between recent use of other antibiotics, PPI or steroids in patients with tCdC or ntCdC, but recent H2-blocker therapy was associated with the development of ntCdC (27.3 *vs*. 0%, *P* = 0.02). In the multivariate analysis, the TLR4 rs1927914 polymorphism (odds ratio [OR] 4.4, 95% confidence interval [CI] 1.6–11.8, *P* = 0.003) and recent cefepime therapy (OR 5.3, 95% CI 2.1–13. *P*<0.001) were independently related to tCdC, and recent therapy with cefuroxime (OR 11.7, 95% CI 2.3–60.2, *P* = 0.003) and intravenous glycopeptide (OR 10.9, CI: 2.1–57.2, *P* = 0.005) were related to ntCdC (Table 4). These results were statistically significant under the Bonferroni correction for multiple testing (*P*<0.01).

**Table 3:** Medications during hospitalization in 357 patients without *Clostridium difficile* colonization (CdC) at admission, stratified by the presence or absence of toxigenic or nontoxigenic CdC in the feces after admission.

Medications	No CdC,	Toxigenic	Nontoxigenic	*P* values		
	n = 321	CdC, n = 25	CdC, n = 11	*a*	b	c
Cephalosporins	223 (69.5)	22 (88.0)	8 (72.7)	0.07	1.00	0.34
Cefazolin, iv	10 (3.1)	1 (4.0)	1 (9.1)	0.57	0.31	0.52
Cefuroxime, iv/o	30 (9.3)	2 (8.0)	4 (36.4)	1.00	0.02	0.06
Ceftazidime or ceftriaxone, iv	179 (55.8)	13 (52.0)	6 (54.5)	0.84	1.00	1.00
Cefepime, iv	56 (17.4)	12 (48.0)	2 (18.2)	0.001	1.00	0.14
Fluoroquinolones, iv/o	17 (5.3)	0 (0)	2 (18.2)	0.62	0.13	0.09
Penicillins other than piperacillin-tazobactam, iv/o	58 (18.1)	7 (28.0)	4 (36.4)	0.28	0.13	0.70
Piperacillin-tazobactam, iv	58 (18.1)	2 (8.0)	2 (18.2)	0.28	1.00	0.57
Carbapenems, iv	88 (27.4)	7 (28.0)	4 (36.4)	1.00	0.51	0.70
Glycopeptides, iv	48 (15.0)	7 (28.0)	5 (45.5)	0.09	0.02	0.45
Metronidazole, iv/o	6 (1.9)	0 (0)	1 (9.1)	1.00	0.21	0.31
Proton pump inhibitors, iv/o	40 (12.5)	4 (16.0)	3 (27.3)	0.54	0.16	0.65
H2-blockers, iv/o	34 (10.6)	0 (0)	3 (27.3)	0.15	0.11	0.02
Steroids, iv/o	73 (22.7)	7 (32.0)	2 (18.2)	0.33	1.00	0.69

Data are expressed as case no. (%). Note: iv = intravenous, o = oral.

a toxigenic CdC *vs.* no CdC;

b nontoxigenic CdC *vs.* no CdC;

c toxigenic CdC *vs.* nontoxigenic CdC.

**Table 4:** Multivariate analysis of risk factors for fecal toxigenic or non-toxigenic *Clostridium difficile* colonization (CdC) during hospitalization among 347 patients. without CdC at admission.

Characters	Toxigenic CdC			Nontoxigenic CdC		
	Oddsratio	95% confidenceinterval	*P* value	Oddsratio	95% confidence interval	*P* value
TLR4 rs1927914 polymorphism, GG type	4.4	1.6–11.8	0.003	0.6	0.1–5.3	0.12
Prior use of cefepime	5.3	2.1–13.2	<0.001	2.1	0.4–14.9	0.47
Prior use of cefuroxime	0.6	0.1–3.1	0.55	11.7	2.3–60.2	0.003
Prior use of glycopeptides	2.1	0.8–5.7	0.16	10.9	2.1–57.2	0.005
Prior use of H2-blockers	0	0	1.00	2.2	0.4–11.0	0.33

## Discussion

Overall, the incidence density of CDAD among the patients with tCdC was at least 10-fold higher than that of those without CdC. The result, which is compatible with previous studies [5], [23], suggests that tCdC is a requisite for CDAD. Though advanced age, prior use of antibiotics, prolonged hospitalization, presence of comorbidities with functional impairment or immune gene (such as IL-8) polymorphism had been shown to be associated with an increased rate of CDAD and disease recurrence [2], [3], [4], the risk factor of tCdC in hospitalized patients had not previously been well investigated.

Patients with CdC were likely to develop CDAD, as evidenced in our and another recent study [24], in which 19 (51.3%) of 37 patients with CdC at time of admission had CDAD and 28 (12.0%) of 234 patients without CdC developed hospital-acquired CDAD (*P*<0.0001). However, an early investigation had concluded that asymptomatic CdC was associated with a lower risk of CDAD [25], but several concerns should be raised for this summary report from four published studies conducted at two hospitals 20–30 years ago (between 1983 and 1993). These four studies were criticized by different study designs, including definitions of diarrhea, hospital settings, calendar years, and follow-up periods. The authors noted that the rate of CDAD among their symptom-free colonized patients was very low, 1.0% (2 of 192 patients). In contrast, 3 (11.5%) of our 26 healthcare-associated tCdC developed CDAD. It is possible that there will be great variation in the virulence of *C. difficile* strains and antibiotic pressure in the study hospitals in the previous and present study, making comparisons between the results of two studies difficult.

Although many classes of antibiotics, including cephalosporins, penicillins, clindamycin, and fluoroquinolones, were known to be linked to the development of CDAD [26], [27], [28], and previous exposure to clindamycin [29], penicillins [29], or fluoroquinolones [30] was linked to CdC in several retrospective studies, a prospective longitudinal study was lacking. In our previous study we identified the patients were more likely to have tCDC if they had received more than one class of antibiotics than if they had received monotherapy^5^. In this prospective study we analyzed the impact of individual antibiotic on CdC and found cefepime use was associated with tCdC, whereas cefepime exposure had not been significantly related to subsequent CDAD in other studies [31], [32], [33].

Although TLR4 has often been linked to Gram-negative infection [12], TLR4 may play a role in innate immunity against *C. difficile,* as evidenced by an animal study in which mice displayed an increased severity of *C. difficile* infection in the absence of TLR4 [34]. To further elucidate the correlation of CdC and TLR4 polymorphisms, we studied two alleles, rs10983755 and rs1927914, located in the 5′ flanking region of the *TLR4* gene [20] because these polymorphisms of the 5′ flanking region may have functional consequences for TLR4 expression or signaling activity [35], [36]. In addition to prior cefepime therapy, we found that only TLR4 rs1927914 (GG genotype) was significantly associated with tCdC. However, the functional relevance of the TLR4 polymorphism and CdC requires further investigation. Ryan *et al.* had reported that TLR4 may play a role in innate immunity against *C. difficile* through recognition of surfacr layer proteins, as evidenced by the increased severity of *C. difficile* infections in TLR4-deficient mice [34]. Moreover, surface layer proteins were antigenically variable between *C. difficile* strains [37], and may be related to strain variation of the interaction of toxigenic or nontoxigenic *C. difficile* strains and TLR4 polymorphism in human hosts.

Colonization with non-toxigenic *C. difficile* has been used to prevent CDAD in piglets [6] and hamsters [7], [38], and the administration of a nontoxigenic *C. difficile* strain has been used in two patients to treat relapsing CDAD [39]. Nevertheless, the evidence that ntCdC prevents CDAD remains insufficient. In our study, there were fewer patients with ntCdC developing tCdC or CDAD, suggesting a protective potential of fecal colonization with nontoxigenic *C. difficile* strains against CDAD. However, 5 (15%) of the 34 patients with ntCdC experienced diarrheal illness that was not related to *C. difficile* and characterized by the absence of toxigenic *C. difficile* in the stool. Another unresolved issue is whether nontoxigenic *C. difficile* isolates can be the cause of diarrhea, as previously suggested [8]. Without exclusion of other microbiological factors as the cause of healthcare-associated diarrhea, it is too early to refer to the nontoxigenic *C. difficile* as the pathogen in the five symptomatic patients.

Though Loo *et al.* had published similar work dealing with risk factors of *C. difficile* infections or colonization [2], their study focused only on healthcare-associated CdC events and their patients with healthcare-associated CdC included the patients with ntCdC and tCdC. The risk factors of ntCdC in hospitalized patients have not been previously reported; this is the first prospective study to reveal that recent treatment with parenteral cefuroxime or glycopeptide was independently associated with ntCdC. However, on the contrary, cefuroxime [40] or glycopeptide [31] use was associated with an increased risk of CDAD in several retrospective studies. These conflicting results may stem from the variable nature of the study designs, patient populations, or study end points. The role of parenteral cefuroxime and glycopeptide use in the subsequent development of tCdC or CDAD warrants further clinical evaluation.

There were some limitations to our study. Firstly, fecal samples were available for microbiological studies only during hospitalization, and thus, the presence or absence of CdC after discharge was unknown and the potential factors predisposing to CdC, such as the use of offending antibiotics, H2-blockers or PPI in the community, would not be detected. Secondly, our study was conducted in an area without recognized spread of hypervirulent ribotype 027 strains, and therefore these results may not be generalizable to western countries. Thirdly, the number of CDAD cases observed was so limited that no significant risk factor for CDADs other than tCdC could be identified. Nevertheless, our study provides narratives of the hospitalized adults with or without CdC and the knowledge of risk factors of tCdC or ntCdC that could be useful in the prevention of CDAD.

In conclusion, in the prospective culture-based surveillance study, the dynamics of fecal colonization by toxigenic and nontoxigenic *C. difficile* isolates were revealed. The individuals with tCdC were more likely to have CDAD than those without CdC, and clinical and host factors associated with ntCdC were identified. The clinical significance of ntCdC remains to be defined.

## Supporting Information

Table S1 **Probe sequences used for detecting TLR2, TLR4 and NF-κB polymorphisms.**
(DOC)
