# Supplementary material for: Risk Factors of Fecal Toxigenic or Non-Toxigenic Clostridium difficile Colonization: Impact of Toll-Like Receptor Polymorphisms and Prior Antibiotic Exposure
Source: PLoS One. 2013 Jul 25;8(7):e69577. doi: 10.1371/journal.pone.0069577 (PMC3723847; doi:10.1371/journal.pone.0069577)
Supplement: Table S1 — Probe sequences used for detecting TLR2, TLR4 and NF-κB polymorphisms. (DOC) [file pone.0069577.s001.doc]

**Table S1.** Probe sequences used for detecting TLR2, TLR4 and NF-κB polymorphisms.

| Gene  (rs number) | Allele | Probes |
| --- | --- | --- |
| TLR2 |  |  |
| rs1898830 | A/G | Probe: 5-ATAGTAAAATAAATCCAGAGAAATC[A/G]GAACAGGGGAAATAATAATATAAGA-3 |
| rs3804099 | C/T | Probe: 5-CAAAAAGTTTGAAGTCAATTCAGAA[C/T]GTAAGTCATCTGATCCTTCATATGA-3 |
| rs7656411 | G/T | Probe: 5-TTTTTAAGCAAATATATACCTAGAG[G/T]TTCCTCATAATGACTCAAAAATAGT-3 |
| TLR4 |  |  |
| rs10983755 | A/G | Probe: 5-TCCCTCACAGCTTGGTTTTTGACAC[A/G]TTGGATTGGAAGTGCTTGGAGGATA-3 |
| rs1927914 | A/G | Probe:5-AGTAGAACTATCTAGGACTTAGCAT[A/G]CATAATATTCCTGTTTTAAATCAGG-3 |
| NF-κB |  |  |
|  | Del  Ins | Primer 1: 5-GCCTCCGTGCTGCCT-3  Primer 2: 5-AGGGAAGCCCCCAGGAA-3  Probe 1: 5-TTCCCCGACCATTGG-3  Probe 2: 5-CCGACCATTGATTGG-3 |
